# Editorial: Gene expression: epigenetic and transcriptional regulation in neurodegenerative diseases and ageing

**DOI:** 10.3389/fnins.2023.1342890

**Published:** 2023-12-12

**Authors:** Vijaykumar Yogesh Muley

**Affiliations:** Instituto de Neurobiología, Universidad Nacional Autónoma de México, Querétaro, Mexico

**Keywords:** neurodegenerative diseases, gene expression and regulation, methylation, cell death, aging, m6A modification, brain

Neurodegenerative Diseases (NDs) constitute a diverse group of over 100 common and rare neurological conditions (Przedborski et al., 2003), characterized by the degeneration of neurons and their intricate circuits (Hardy and Gwinn-Hardy, 1998; Wilson et al., 2023). Despite significant advancements, the origins of NDs remain elusive, with aging recognized as a pivotal risk factor (Hou et al., 2019), along with genetic variations, environmental exposures, and lifestyle choices (Wyss-Coray, 2016). Collectively, these factors influence gene expression through both epigenetic and transcriptional regulation. This Research Topic aims to deepen our understanding of gene expression and regulation in NDs and aging, featuring eight articles on data resources, epigenetics, cell death, metabolism, and the gut-microbiome axis.

The NCBI Gene Expression Omnibus (GEO) serves as a crucial repository, housing 64,653 microarray and transcriptomic datasets for humans as of November 2023 (Barrett et al., 2011). While invaluable, accessing and analyzing thematic data poses challenges. In response, data mining and curation studies have proven instrumental. Li et al. introduce the SCAD-Brain database, consolidating Alzheimer's disease (AD) and mild cognitive impairment transcriptomic data from 17 single-cell RNA-Seq (scRNA-Seq) projects sourced from the GEO and Synapse databases. SCAD-Brain encompasses 21 datasets comprising 10 distinct brain regions, 16 primary cells, and 1,564,825 individual cells. Researchers can utilize SCAD-Brain for diverse analyses, including cell diversity, cell marker identification, differential expression, and functional enrichment across brain regions and cell types. It is user-friendly for bench scientists with minimal programming experience, although it's worth noting that these functionalities contribute to a notably slow response from the SCAD-Brain server. In a complementary study, Abdullatef and Farina conducted a comprehensive review of *ex-vivo* human and mouse central nervous system transcriptomic studies indexed in PubMed. They cataloged and categorized around 100 transcriptomic datasets spanning human and mouse development, aging, and five major NDs, including AD, Parkinson's (PD), Huntington's, Multiple sclerosis, and Amyotrophic lateral sclerosis. Researchers proficient in computational analysis may find these listed datasets valuable.

Epigenetic processes involve molecular switches influenced by development, environmental factors, and experiences, regulating gene expression without altering DNA and impacting traits across generations (Jangid et al., 2018). A pivotal mechanism involves the addition or removal of methyl groups from DNA or RNA, orchestrated by methyltransferases and demethylases (Traube and Carell, 2017). Methyltransferase-like enzymes, such as METTL3, play a crucial role in depositing methyl groups specifically on the nitrogen atom at the sixth position of the adenine base in RNA, forming N6-methyladenosine (m6A; Masatoshi et al., 2018). This widespread and reversible RNA modification influences RNA stability, splicing, translation, and subsequently, protein production (Adams and Cory, 1975; Fu et al., 2014), and have relevance in various diseases, including cancer, neurodevelopmental, and metabolic diseases (Shafik et al., 2021). Methylated RNA Immunoprecipitation Sequencing (MERIP-Seq) is a powerful technique for transcriptome-wide mapping of RNA methylation, with a primary focus on m6A modification (Dominissini et al., 2012).

In a comprehensive study, Hu et al. used MERIP-Seq and RNA-Seq methodologies to investigate genome-wide m6A RNA modifications and transcription profiles in the mouse hippocampus at different developmental stages: postnatal 10 days (P10), 11 weeks (adult), and 64 weeks (aged). The hippocampus, a vital seahorse-shaped brain region responsible for memory consolidation and spatial navigation, underwent spatial cognition and learning impairment in aged mice, as demonstrated by the Barnes maze test. Further analysis revealed differential high expression and m6A methylation of PD-1/PD-L1 pathway genes (Pdcd1, Myd88, and Ptpn6), potentially contributing to cognitive dysfunctions in the aged hippocampus. Additionally, the ectopic expression of Mettl3 was shown to affect the expression of PD-1/PD-L1 pathway genes, leading to a significant spatial cognitive deficit. Their data suggest that m6A mediated by METTL3 contributes to cognitive deficits linked to the hippocampus through the PD-1/PD-L1 pathway in aged mice. It's crucial to note that PD-1/PD-L1 pathway activation has also been shown to protect against inflammatory processes associated with neurodegeneration (Kummer et al., 2021) and autoimmune diseases (Nishimura et al., 2001), while suppressing them in cancers (Yamaguchi et al., 2022; Luke et al., 2023). Therefore, the observed effects of ectopic expression of METTL3 on the activation of the PD-1/PD-L1 pathway in aged mice warrant further investigation.

Shifting focus to DNA methyltransferase 1 (DNMT1), responsible for maintaining DNA methylation marks postnatally (Lyko, 2018), Wang et al. investigated genetic variations in DNMT1 among 712 sporadic PD patients and 696 controls in the Chinese population. The study identified the protective allele rs9305012 against sporadic PD, with reduced DNMT1 expression as observed in the postmortem PD brains. Additionally, potential regulatory effects of rs9305012 on P2RY11 gene expression were noted. However, the functional impact of rs9305012 remains uncertain. Nevertheless, genetic variants of DNMT1 and P2RY11 have been associated with narcolepsy in patients with cerebellar ataxia and cataplexy, and consistently, both genes have been expressed in the cerebellum, the region usually not associated with PD, but involved in movement and reward, which may have relevance in the motor dysfunctions in PD (Kornum et al., 2011; Pedroso et al., 2013).

Over 30 cell death modalities have been proposed in living organisms, a subject open to debate due to the often-blurred distinction caused by cross-talk and overlapping molecular players and features in various cell death pathways (Galluzzi et al., 2018; Liu et al., 2018). It is not surprising that identifying the molecular mechanisms underlying progressive neuronal loss in NDs is challenging.

In a study by Tan et al. differentially expressed mRNAs and lncRNAs in AD were identified using GEO microarray datasets. Through various bioinformatics approaches, authors identified lncRNAs that compete with miRNAs, post-transcriptionally regulating differentially expressed mRNAs associated with ferroptosis—a recently discovered iron-dependent death mode (Galluzzi et al., 2018). These lncRNAs are called competitive endogenous RNAs (ceRNAs), which sequester miRNAs by forming complementary bindings with them affecting the translation of their target mRNAs (Thomson and Dinger, 2016). The authors propose that the five ferroptosis-associated genes (EPT1, KLHL24, LRRFIP1, CXCL2, and CD44) differentially expressed in AD are regulated by a network of 28 ceRNAs and their 110 miRNA targets. Further, experimental validation is required to confirm these findings.

In another study, Wei et al. identified necroptosis-related genes differentially expressed in AD brains compared to control samples. Necroptosis is a programmed version of necrosis (Galluzzi et al., 2018). Authors developed a five-gene diagnostic model based on ACAA2, BHLHB4, CACNA2D3, NRN1, and TAC1 expression in AD using machine learning approaches, showing outstanding diagnostic performance closely related to AD's pathologic hallmarks. While this model may aid in understanding disease heterogeneity and neuronal loss mechanisms in AD, it needs thorough comparison with existing markers implicated in AD diagnostics.

Furthermore, Guo et al. analyzed three publicly available single-cell and bulk RNA-Seq transcriptomic datasets from brain and blood samples of AD patients and age-matched controls based on the metabolic activity of 118 regulators of glutamine metabolism using an array of bioinformatics and machine learning methods. Nine glutamine metabolic-associated genes were selected based on differential expression to develop a risk score for AD diagnosis, which has shown higher accuracy than classical clinical assessment. Among the nine genes, PHF1 may play a crucial role in glutamine metabolism in AD, as demonstrated through *in-vivo* and *in-vitro* experiments. It appears that PHF1 knockdown reduces glutamine biosynthesis and protects against neurite loss and cell injury in AD neurons.

Finally, Zeng et al. analyzed public data on a genome-wide association study of gut microbiota of 18,340 individuals, 63,926 AD patients and 10,528,610 controls using Mendelian randomization to explore the causal relationship between gut microbiota and the risk of AD. Members of Actinobacteria were associated with a higher AD risk, while Ruminococcus showed a protective effect. Further research is needed to confirm these causal relationships.

In summary, the research article featured in this Research Topic provides essential resources, a conceptualized bioinformatics approach, and identifies crucial gene regulation mechanisms operational in NDs, enhancing our understanding of the subject. The common theme that emerges from these studies is that the genes directly or indirectly associated with inflammation, auto-immunity, and immune responses are differentially expressed in neurodegenerative diseases and aging.

## Author contributions

VM: Writing – original draft, Writing – review & editing.
